# Andrographolide exerted anti-inflammatory effects thereby reducing sex hormone synthesis in LPS-induced female rats, but had no effect on hormone production in healthy ones

**DOI:** 10.3389/fphar.2022.980064

**Published:** 2022-09-15

**Authors:** Xiaoyan Yuan, Wenhao Xu, Zijun Yan, Xingmeng Xu, Yanqing Chen, Simin Chen, Ping Wang

**Affiliations:** ^1^ Panzhihua Central Hospital, Panzhihua, China; ^2^ College of Pharmacy, Chengdu University of Traditional Chinese Medicine, Chengdu, China

**Keywords:** andrographolide, inflammation, metabonomic test, steroid hormone biosynthesis pathway, steroidogenic acute regulatory protein

## Abstract

Females have higher inflammatory tolerance because they have some special sex-related anti-inflammatory pathways. Andrographolide, a diterpene lactone compound from *Andrographis paniculata* (Burm.f.) Nees, has a powerful anti-inflammatory effect. But whether andrographolide regulates sex-related anti-inflammatory pathways in females has yet to be reported. A non-targeted metabonomics method was employed to investigate the metabolic pathways of andrographolide in LPS-induced inflammatory female rats. Substances and genes were then selected out of gender-related pathways discovered by metabonomics experiments and their quantities or expressions were evaluated. Furthermore, the effects of andrographolide on these chemicals or genes in non-inflammatory female rats were also examined in order to investigate the cascade interaction between anti-inflammatory mechanisms and metabolites. The biomarkers of 24 metabolites in plasma were identified. Following pathway enrichment analysis, these metabolic markers were clustered into glycerophosphate, glycerolipids, inositol phosphate and steroid hormone synthesis pathways. Validation experiments confirmed that andrographolide lowered post-inflammatory female sex hormones such as progesterone, estradiol, corticosterone, and testosterone rather than increasing them. Andrographolide may have these effects via inhibiting the overexpression of CYP11a1 and StAR. However, andrographolide had no effect on the expression of these two genes or the four types of hormones in non-inflamed female rats. Similarly, andrographolide decreased TNF-α, IL-6 and IL-1β production in inflammatory rats but showed no effect on these inflammatory markers in non-inflammatory rats. LPS and other inflammatory cytokines promote hormone production, which in turn will prevent increased inflammation. Therefore, it may be hypothesized that andrographolide’s reduction of inflammatory cytokine is what generates its inhibitory action on sex hormones during inflammation. By blocking the activation of inflammatory pathways, andrographolide prevented the stimulation of inflammatory factors on the production of sex hormones. It does not, however, directly inhibit or enhance the synthesis of sex hormones.

## 1 Introduction

**FIGURE 1:**
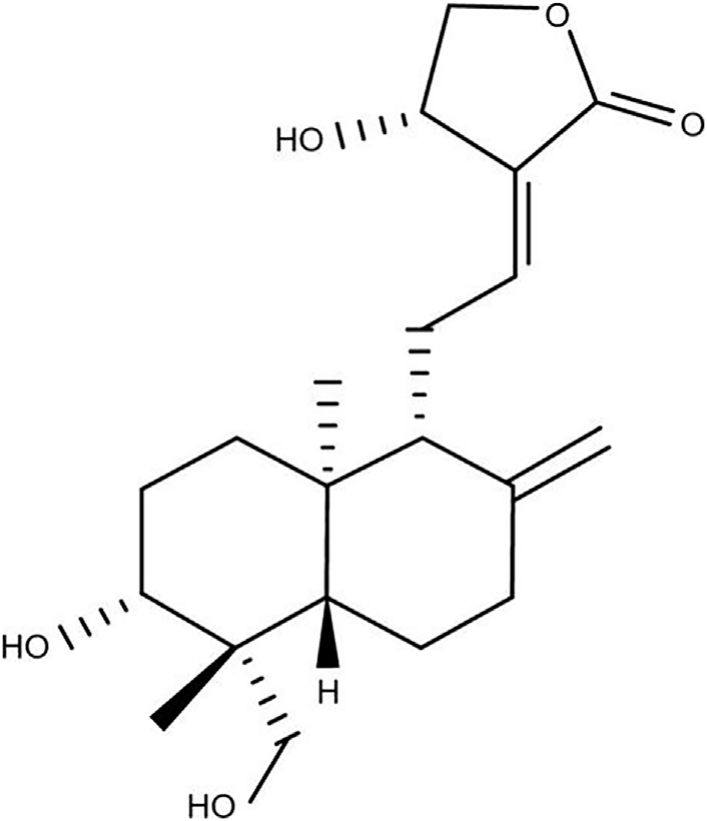
Chemical structure of Andrographolide (AG).

Andrographolide (AG, Figure 1) is a diterpene lactone compound and widely used in the treatment of infectious inflammation and other diseases (Yan et al., 2013), which is an major active component of *Andrographis paniculata* (Burm.f.) Nees, a traditional herb medicine using in China, India and Sri Lanka. AG has many pharmacological effects, among which the anti-inflammtory effect has drawn more attention. In many *in vivo* experiments, it has been reported that AG may alleviate inflammation caused by different stimuli by regulating the activation of various signal pathways.

AG protects male BALB/c mice from acute lung injury induced by lipopolysaccharide (LPS), which may be related to andrographolide’s significant inhibition of phosphorylation levels of IKKβ, IκBα and nuclear factor-κB (NF-κB) p65 (Zhu et al., 2013). And it was also found to decrease the activation of nuclear factor κB signal induced by Poly I:C in male BALB/c mice pneumonia model, but did not inhibit IRF3-mediated immune response (Cui et al., 2020). Similarly, AG significantly reduces the level of mouse cortical chemokines induced by LPS also by inhibiting the activation of NF-κB or c-Jun N-terminal kinase (JNK) (Wong et al., 2016). When treating complete Freund’s adjuvant-induced arthritis, AG inhibits a series of molecules related to arthritis, such as cyclooxygenase-2 (COX-2), NF-κB, p-p38, CD40, TNF-α, IL-1β, and IL-6 (Gupta et al., 2020). Meanwhile, accumulative studies have been designed to assess AG’s anti-inflammatory effect *in vitro*. In Raw 264.7 macrophages, AG inhibited the LPS-induced inflammatory response, which was attributed to the blockage of the NF-κB and MAPK pathway signal transduction and reduced pro-inflammatory factor release (Li et al., 2017). In the LPS-induced bovine endometrial epithelial cell, AG activated the nuclear factor erythroid 2-related factor 2 (Nrf2) pathway by inhibiting Kelch-like ECH-associated protein 1 (KEAP1) and upregulating the expression of Nrf2 mRNA to inhibit the release of pro-inflammatory factors (Fu et al., 2021). The reseach also reported AG inhibited NLR family pyrin domain containing 3 (NLRP3) inflammasome and its downstream targets, such as casp-1 (p20) and IL-1β(Ahmed et al., 2021; Lo et al., 2021).

Early studies have found that women’s tolerance and survival rate in sepsis caused by Gram-negative bacteria are significantly better than men’s (Zellweger et al., 1997; Drechsler.,et al., 2012). In LPS-induced sepsis rats, it was also observed that female mortality was lower than that of male (Kosyreva,et al., 2018). This may be due to the possibility that X-linked genes cause an abnormal gender gap in the risk of immune-mediated illnesses (Stein,et al., 2021). Another important reason may be the high level of special gonadal hormones in female, which has certain anti-inflammatory effects (Kosyreva,et al., 2018). It may also be based on these consideration, in order to avoid the influence of estrogen on the evaluation of anti-inflammatory activity, most of the *in vivo* experiments reported so far use male animals, such as many AG anti-inflammatory experiments mentioned above. In addition, the interaction between drugs and sex hormones could not be observed in cell experiments *in vitro*. Therefore, it is intriguing to consider if AG has a unique mechanism that has an anti-inflammatory impact on females.

**FIGURE 2:**
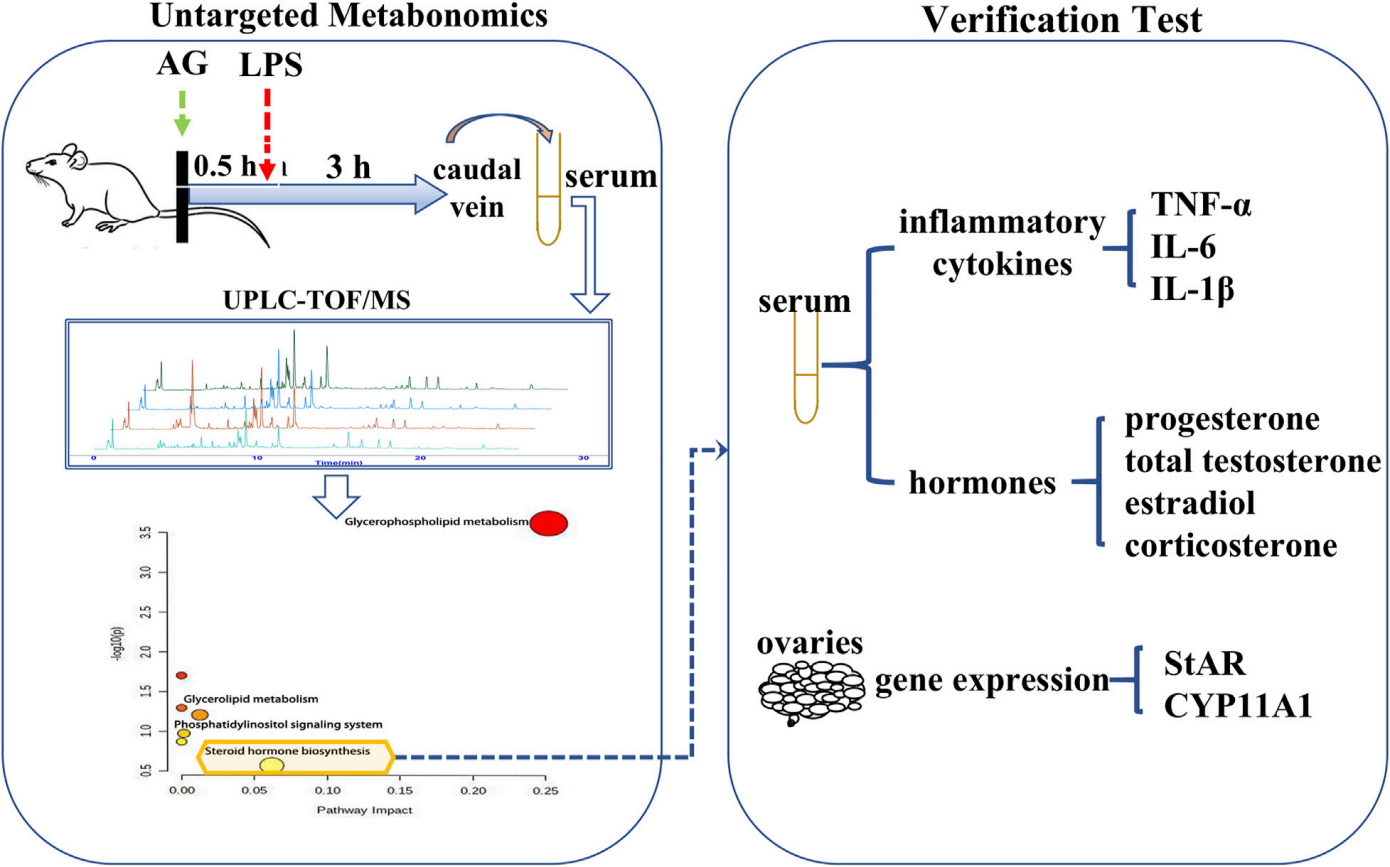
Schematic diagram of tests process.

Non-targeted metabonomics was employed to investigate the metabolic pathways of AG in LPS-induced inflammatory female rats. The contents or expression levels of several chemicals and genes were then identified from the gender-related pathways. In order to investigate the cascade link of anti-inflammatory mechanisms, it was also assessed how AG affected these substances and genes in uninflamed female rats. The research procedure is depicted in Figure 2.

## 2 Materials and methods

### 2.1 Materials

Andrographolide (AG>99%) was purchased from Chengdu Manster Biotechnology Co. Ltd. (Chengdu, China). Lipopolysaccharide (LPS) was purchased from Sigma-Aldrich Chemical Co. (Shanghai, China). Sodium carboxymethyl cellulose (CMC-Na) and formic acid were supplied by Chengdu Kelon Chemical Reagent Factory (Chengdu, China). Other reagents used in liquid chromatography were of chromatographic grade. Tumor necrosis factor-α (TNF-α), Interleukin-6 (IL-6), Interleukin-1β (IL-1β) ELISA kits were obtained from Hangzhou Lianke biotechnology Limited Ltd. (Hangzhou, China). Corticosterone, total testosterone, estradiol, and progesterone ELISA kits were all purchased from Elabscience Biotechnology Co. Ltd. (Wuhan, China).

### 2.2 Animal

Adult female SD rats in SPF grade (weighing 220–240 g) were provided by Spefer Biotechnology Co. Ltd. (Beijing, China). All rats were housed in the standard rat cage on a 12 h light/dark cycle from 9:00–21:00, at a temperature of 22°C with 60% humidity, and free access to standard food and water. Rats were adaptively maintained for at least 4 days, and beginning on the second day, the anal temperature was monitored twice daily for 3 days. The process on rats is strictly in accordance with the Guidelines for Nursing and Use of Experimental Animals compiled by the National Academy of Sciences, so as to minimize the discomfort and pain of animals. This research project was approved by the Animal Ethics Committee of Chengdu University of Traditional Chinese Medicine.

### 2.3 Metabonomic experiment

#### 2.3.1 Determination of the phase of the estrous cycle

The estrous cycle of adult female rats was ascertained using vaginal smears stained with Romanovschi-Giemsa (Kosyreva et al., 2018). The rats used in the subsequent studies were all in a stable diestrus phase, which coincided with the estrous cycle’s lowest blood estradiol concentration.

#### 2.3.2 Metabonomic grouping, model establishment and administration

Female rats (*n* = 24) were randomly divided into four groups. The AG was freshly suspended in 0.5% CMC-Na solution. Rats in low dose group (L) and high dose group (M) were orally administered with AG 20 mg/kg and 40 mg/kg respectively, whereas rats in the normal (N) and model (M) groups were only given CMC-Na solution without AG by gavage. After oral administration for 0.5 h, rats in L, H and M groups received LPS 100 μg/kg by tail vein injection, group received an intravenous injection of saline. Then, 3.0 h after the LPS injection, blood samples were taken from the tail vein. Serum was separated from each blood sample by centrifugation at a speed of 3,500 rounds per minute (rpm) for 10 min. These serum samples were immediately stored at −80°C until the metabolomics analysis. And after LPS injected, anal temperature of rats was measured at 0.5, 1, 1.5, 2, 3, 4, 5, 6, 7, 8, and 12 h respectively.

#### 2.3.3 Sample collection and preparation

Protein precipitation was carried out by adding 100 μl acetonitrile to 50 μl serum. The mixture was vortexed for 60 s, then centrifuged at 13,500 rpm at 4°C for 15 min. Finally, the supernatant was transferred to an auto-sampler vial for further determination.

#### 2.3.4 UPLC-Q-TOF/MS analysis

All the LC/MS data were obtained by 1260 Series Rapid Resolution Liquid Chromatography system (Agilent, United States) and Micro-TOF-QⅡ(Bruker, German). Chromatographic separation is accomplished by a Zorbax Eclipse Plus C_8_ Rapid resolution HT column (3.0 mm × 100 mm, 1.8 μm, Agilent, United States). Water and acetonitrile, each containing 0.1% formic acid, were used as mobile phase A and B respectively. The flow rate of 0.5 ml/min at 30°C was used in linear gradients as follows: 95–88% A (0–3 min), 88–25% A (3–20 min), 25–10% A (20–22 min), 95% A (22–25 min). The standard positive ion mode was selected under the following conditions: full scan range, 50–1200 m/z; data recording period: 0–26 min; drying gas flow: 6 L/min; drying gas temperature: 250°C; nebulizer pressure: 1.0 bar; capillary voltage: 5 kV; 3 μl sample was injected into the UPLC system.

#### 2.3.5 Identification of potential biomarkers

The raw mass spectrometry data were output to MZmine 2.49 to obtain a comprehensive matrix composed of ions and their intensity in all samples (T. Pluskal, et al., 2010). Then, using this matrix, a series of multivariate data analysis methods, including the principal component discriminant analysis (PCA-DA), partial least-squares discriminant analysis (PLS-DA), orthogonal partial least-squares discriminant analysis (OPLS-DA) (Edoardo, et al., 2013) and unsupervised cluster analysis (Yu, et al., 2017), were carried out to get the anti-inflammatory tendency of AG and to find the potential biomarkers. The information of potential biomarkers was obtained by searching databases including KEGG (http://www.genome.jp/kegg/), HMDB (http://www.hmdb.ca/), METLIN (http://metlin.scripps.edu/), LIPIDMAPS (http://www.lipidmaps.org/), PubChem (http://pubchem.ncbi.nlm.nih.gov/) and ChemSpider (http://www.chemspider.com/). The identified metabolites were enriched by metaboanalyzer 3.0 (Pang, et al., 2020).

### 2.4 Verification experiment

#### 2.4.1 Grouping and sample collection

To avoid using too many rats, verification experiments were conducted only in four experimental groups: normal group (6, N), normal with AG administration group (6, NAG), model group (6, M) and high dose (6, H) groups. The procedures for N, M and H groups of rats were exactly the same as those for the corresponding groups in the metabonomic experiments, while NAG group received sequential oral administration of AG 40 mg/kg and intravenous administration of saline. Rats were euthanized with 3% sodium pentobarbital 3.0 h after the LPS injection. Laparotomy was performed to collect blood from abdominal aorta and to separate the ovary. Serum from each blood sample were isolated by centrifugation at 3,500 rpm for 10 min. The samples were immediately stored at −80 °C until the ELISA kits tests were performed.

#### 2.4.2 Real time polymerase chain reaction

The expression of two key enzymes of the enrichment pathway in metabolomics experiments was determined at the gene level by quantitative real-time polymerase chain reaction (qRT-PCR). Total RNA was extracted from ovary with total RNA isolation kit (Foregene biotechnology Ltd., Chengdu, China), and then reverse the total RNA to cDNA with RT EasyTM II kit (Foregene biotechnology Ltd., Chengdu, China). Quantitative real-time PCR performed using the Real Time PCR EasyTM-SYBR Green I kit (Foregene biotechnology Ltd. Chengdu, China) on a PCR machine (Applied Biosystems, Canada).

The expression of selected genes include steroidogenic acute regulatory protein (StAR) and cytochrome CYP11A1 enzyme (P450scc) in the ovary. Primer sequences were obtained from Sanggon Corporation (Shanghai, China) in Table 1. Reactions were performed in duplicate. β-Actin was selected as internal controls. To compute the relative amounts of target mRNA in the samples, the 2^−△△CT^ method was used.

**TABLE 1:** Primer sequences used for RT-PCR.

Gene name		Primer sequences (5′-3′)	Tm (°C)	Product length (bp)	
StAR	F	GCT​GTA​CCA​AGC​GTA​GAG​GT	59.47	103
	R	GGA​CCG​TGT​TCA​GCT​CTG​ATG	61	
CYP11A1	F	TCC​TCT​ACC​AAC​AGT​CCT​CGA​T	60.03	168
	R	GTT​GCC​CAG​CTT​CTC​CCT​GTA	62.06	
Beta-actin	F	GGA​CCT​GAC​AGA​CTA​CCT​CA	51.4	230
	R	GTT​GCC​AAT​AGT​GAT​GAC​CT	52.1	

#### 2.4.3 Determination of corticosterone, total testosterone, estradiol, progesterone, TNF-α, IL-6, and IL-1β concentrations in serum

Metabolomics experiments revealed that LPS significantly stimulates progesterone biosynthesis, while AG inhibits its production. ELISA kits were used to measure blood corticosterone, total testosterone, estradiol, and progesterone in order to confirm the effects of LPS and AG on the pathways that produce steroid hormones. Samples were tested according to the kits manufacturer’s instructions. Serum TNF-α, IL-6 and IL-1β concentrations were determined by ELISA kits too. All tests were performed according to the kit instructions.

### 2.5 Statistical analysis

All results were presented as the mean ± SD. Data were analyzed using One-way analysis of variance (ANOVA) for significance comparison. Values of *p* < 0.05 were considered statistically significant. The correlation analysis was conducted by “PerformanceAnalytics 2.0.4” package (Peterson and Carl., 2020) in R 4.2.1 (R core team, 2022).

## 3 Results

### 3.1 Antipyretic action

**FIGURE 3:**
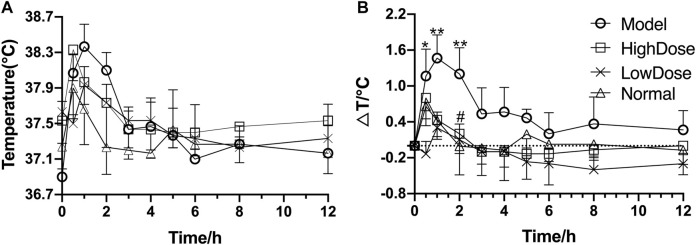
Antipyretic effect of andrographolide on endotoxin-induced inflammation in rats **(A)** temperature figure; **(B)** relative basal temperature difference change figure); ^*^ *p* <0.05 or ^**^ *p* <0.01 versus Normal group and ^#^ *p* <0.05 or ^##^ *p* <0.01 versus Model group by ANOVA.

After injection of lipopolysaccharide for 2 h, the temperature of rats began to rise rapidly with an increase of more than 1.6°C. Andrographolide significantly inhibited the increase of temperature induced by inflammation, and reached its maximum antipyretic effect at 3 h after LPS injection (Figure3). However, the antipyretic effects of high dose AG were the same as that of low dose.

### 3.2 Validation of UPLC-Q-TOF/MS conditions

**FIGURE 4:**
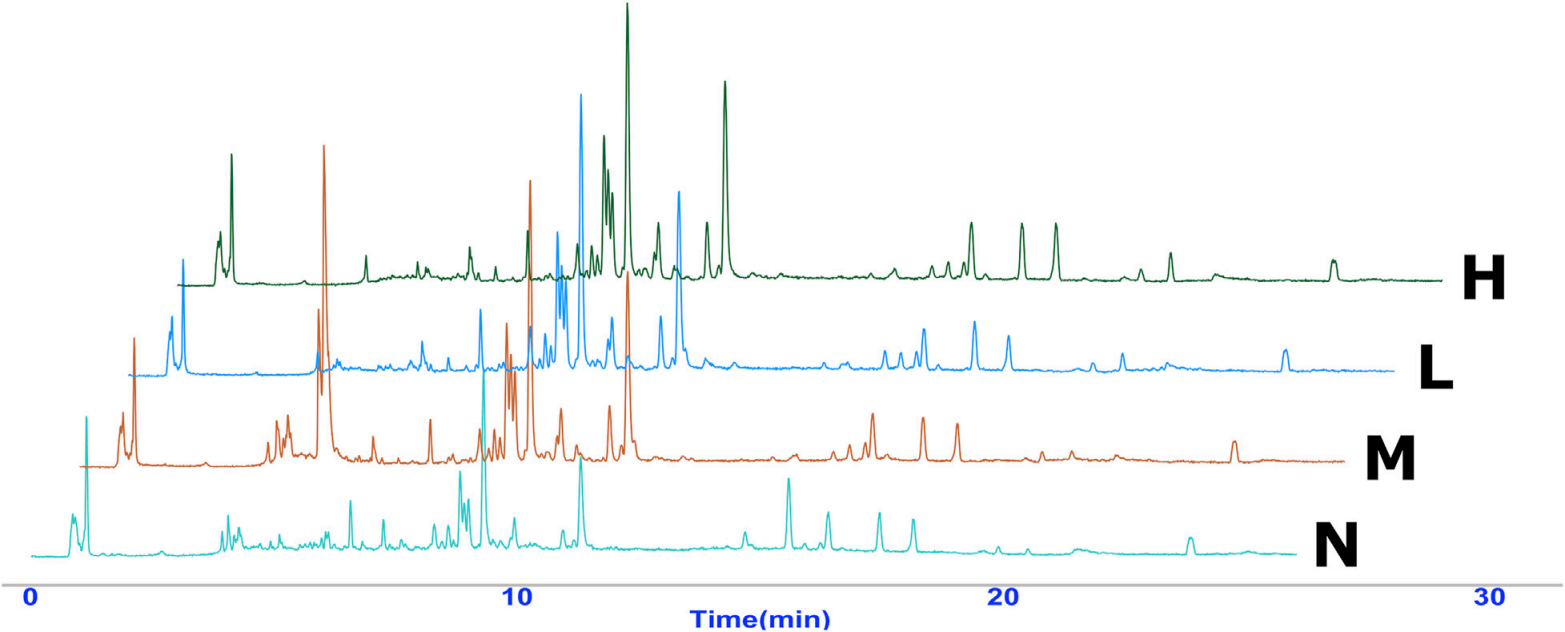
Overlaid representative UPLC-Exactive Plus Orbitrap Mass TIC chromatograms (positive ion) of the serum samples from the four groups, including normal (N), model (LPS 100 μg/kg, M), low dose group (LPS 100 μg/kg + AG 20 mg/kg, L) and high dose (LPS 100 μg/kg + AG 40 mg/kg, H) group.

Typical UPLC-Q-TOF/MS TIC chromatograms of serum samples are shown in Figure 4. Precision, reproducibility, and system stability were confirmed prior to the analysis of experimental serum samples. The results showed that this method could meet the requirements of metabonomics analysis of the subsequent samples.

### 3.3 Pattern recognition and identification of potential biomarkers

**FIGURE 5:**
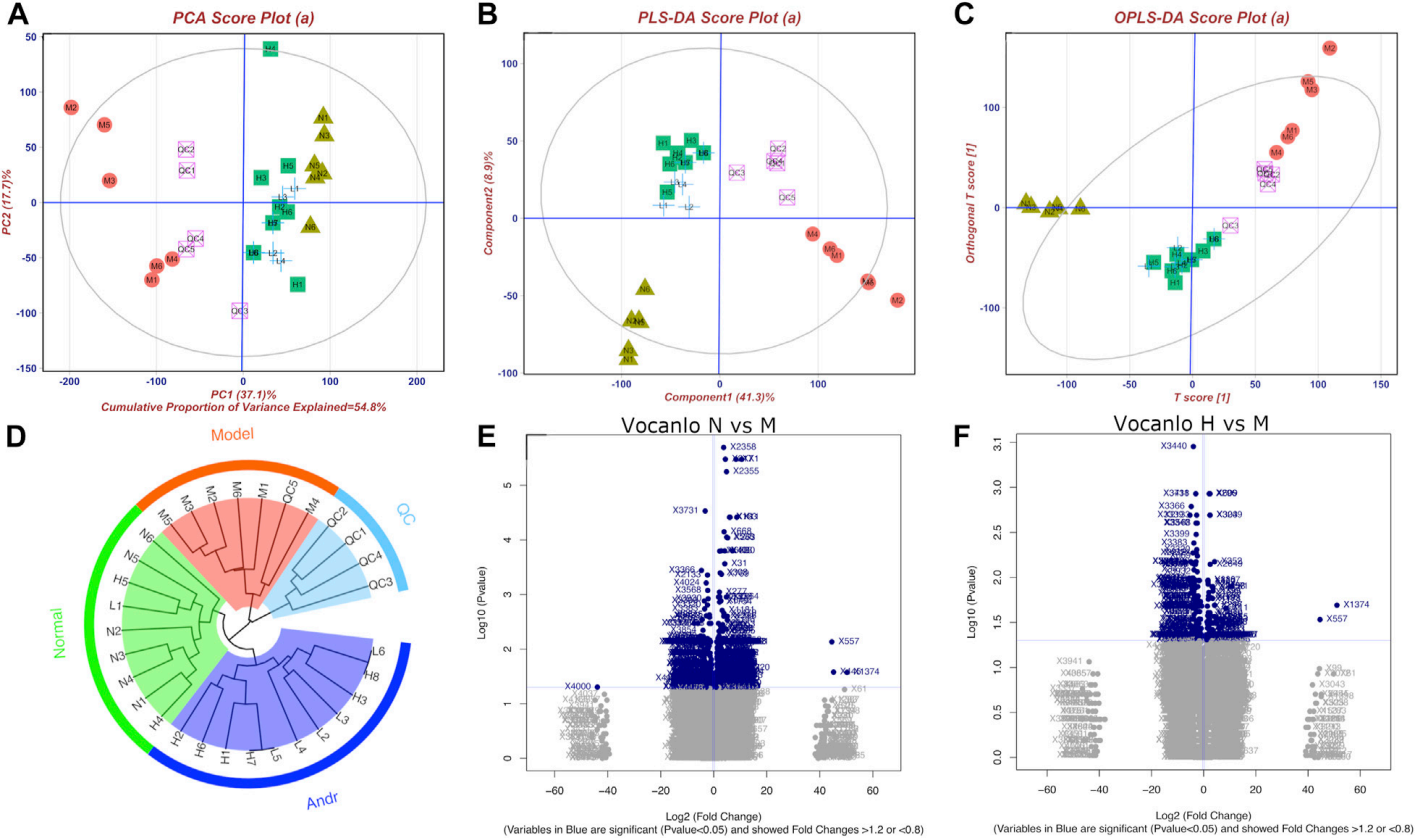
Metabonomics pattern recognition, metabolite identification and pathway enrichment **(A)**: PCA diagram; **(B)** PLS-DA diagram; **(C)** Opls-DA diagram; **(D)** cluster analysis diagram; **(E)** and **(F)** are Volcanic diagram of peak abundance of mass-to-charge ratio chromatography in normal vs. model group and high dose vs. model group, respectively). There are four groups including normal group(N), model group (LPS 100 μg/kg, M), low dose group (LPS 100 μg/kg + AG 20 mg/kg, L) and high dose (LPS 100 μg/kg + AG 40 mg/kg, H) group, and the number represents the ID in the group.

According to the pattern recognize plots (Figures 5 A,B,C), normal and model rats could be successfully distinguished from each other, indicating that the activation of LPS dramatically altered the serum metabolic fingerprint. Furthermore, by administering AG, the model rats’ biochemical abnormalities were gradually reversed to normal. Similar results were obtained through unsupervised cluster analysis (Figure 5D). The findings imply that AG is considerably effective in reducing metabolic abnormalities in LPS-induced inflammatory rats.

**FIGURE 6:**
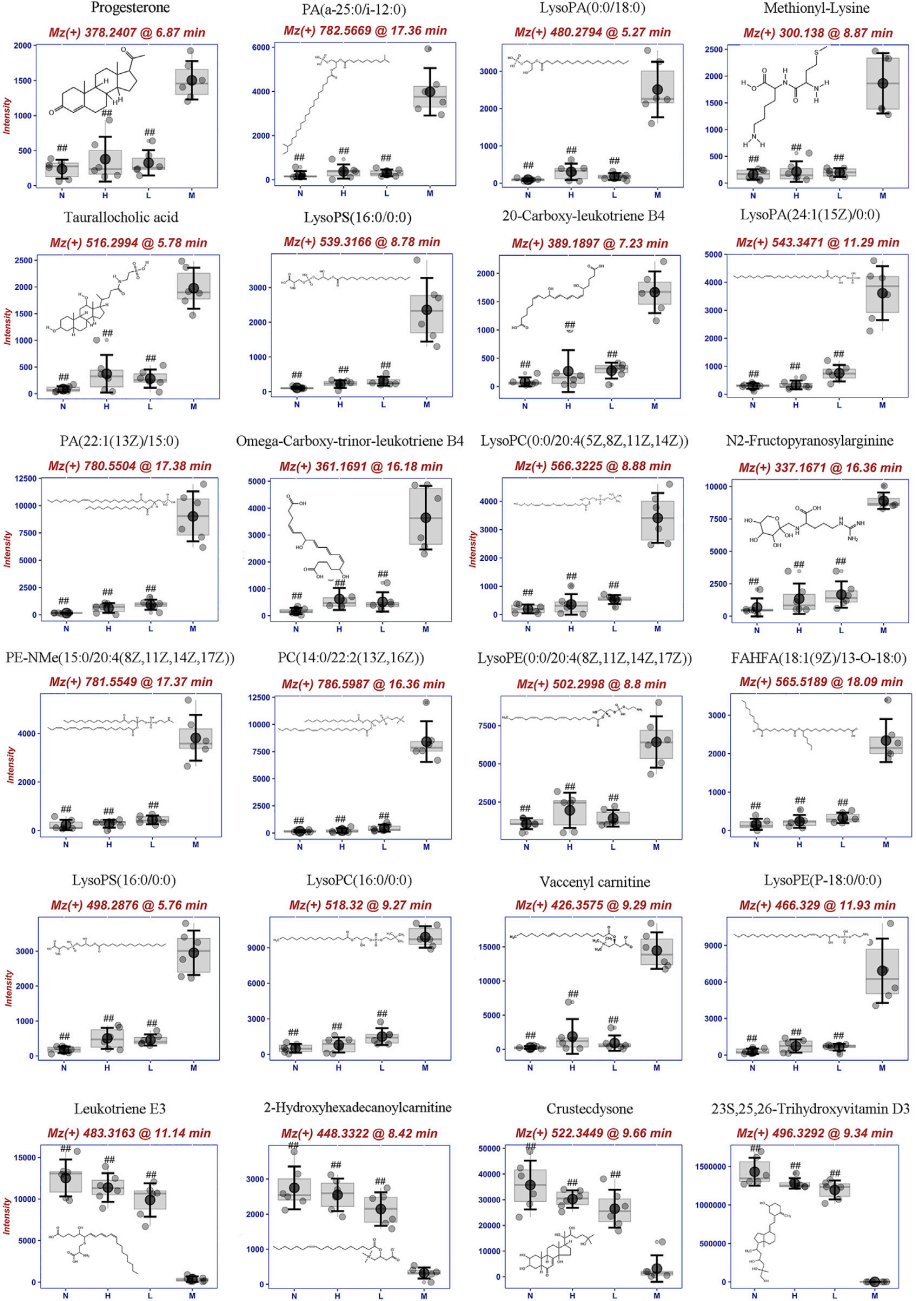
Metabolite biomarkers including structure, mz, retention time and its concentrion in four groups: normal (N), model (LPS 100 μg/kg, M), low dose group (LPS 100 μg/kg + AG 20 mg/kg, L) and high dose (LPS 100 μg/kg + AG 40 mg/kg, H) group. ^#^ *p* <0.05 or ^##^ *p* <0.01 versus Model group by ANOVA.

**FIGURE 7:**
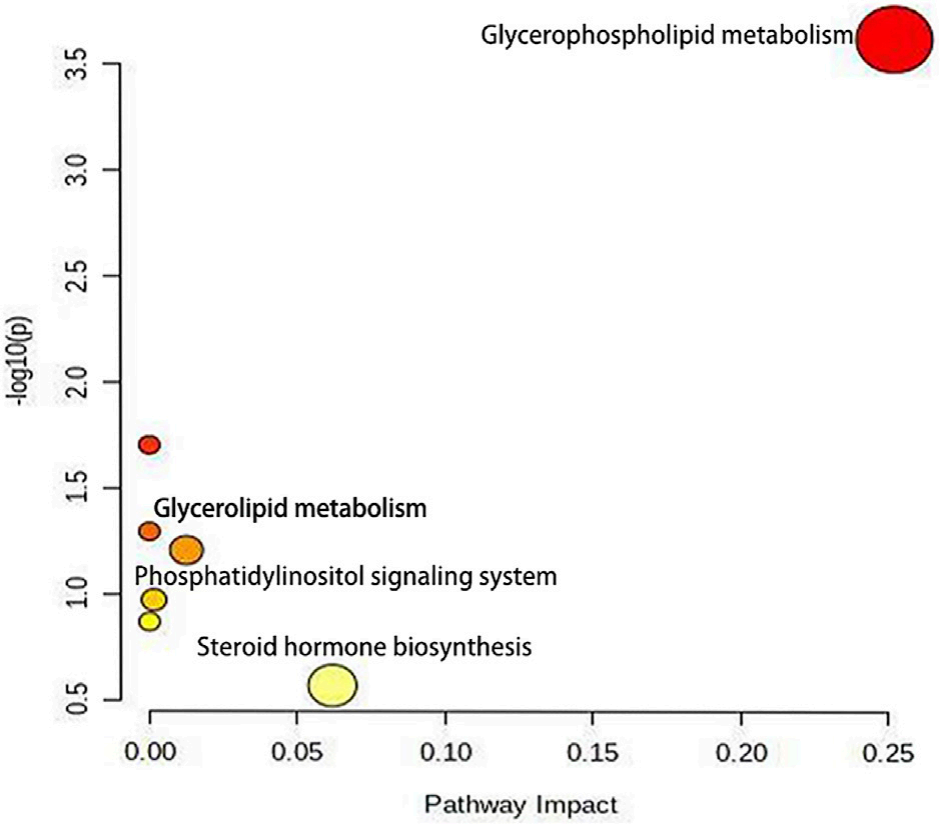
The metabolic pathway enrichment map showed that after lipopolysaccharide induced female rats, glycerophosphate and steroid hormone pathways were more affected by AG.

Furthermore, 24 potential metabolite biomarkers were identified (Figures 5E,F) and their functional pathways were evaluated. Figure 6 displays box plots of the biomarker intensities in rat serum. Progesterone and bile acid, as well as certain lipids like LysoPA, LysoPE, and PC, were all markedly elevated in inflammatory model rats, indicating dysregulation of steroid hormone and glycerophospholipid biosynthesis metabolism (Figure 7) in LPS-induced inflammatory rats. When compared to the normal group, the model group’s biomarker levels were significantly higher (*p* < 0.01).

### 3.4 Effect of andrographolide on sex steroid levels in the serum

**FIGURE 8:**
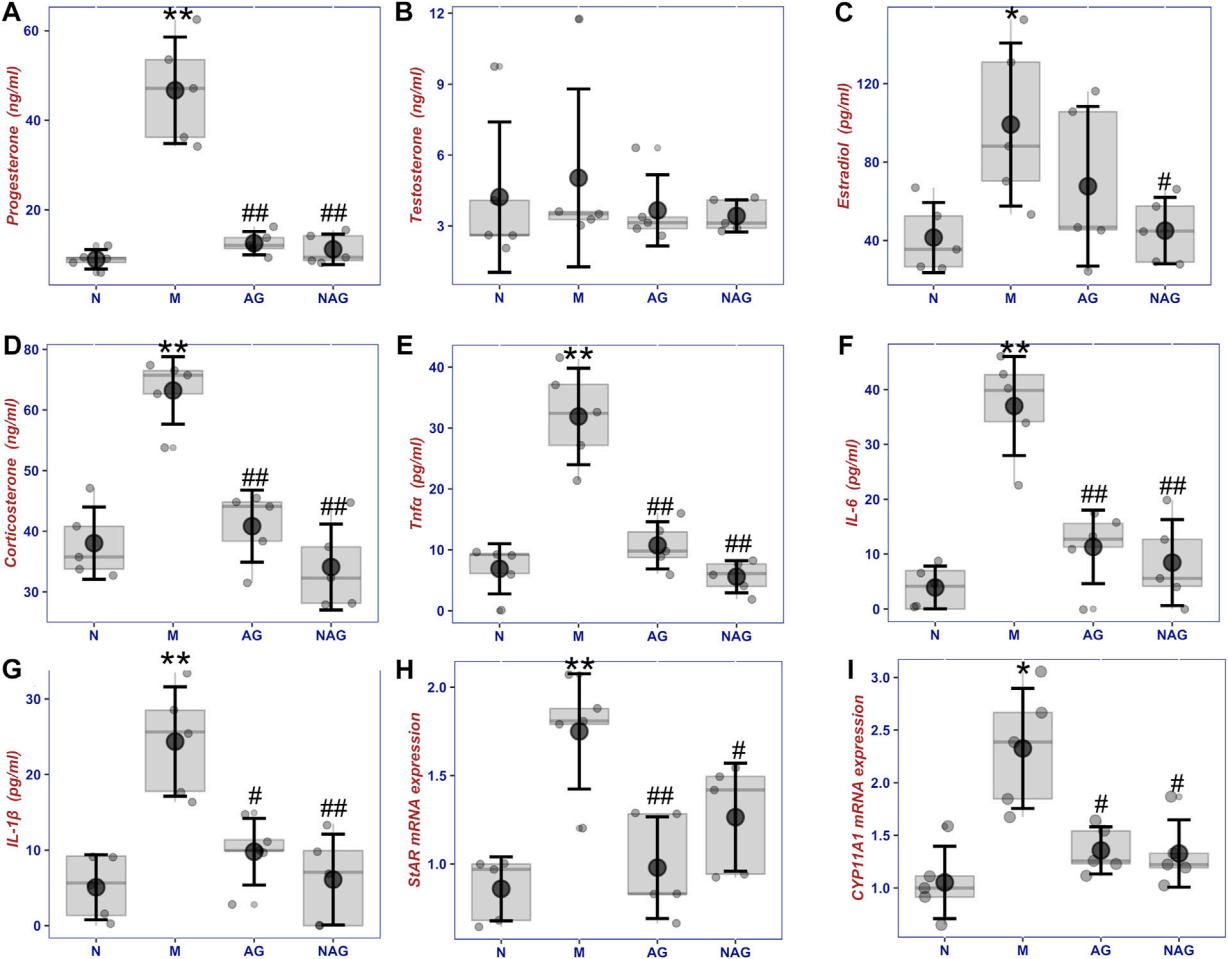
The box plot of sex steroids including progesterone **(A)**, testosterone **(B)**, estradiol **(C)** and corticosterone **(D)**, inflammatory cytokins including TNF-α **(E)**, IL-6 **(F)** and IL-1β **(G)** concentrations in serums, and the sex hormone synthesis gene expression **(H)** and **(I)** in ovories in normal group (6, N), normal with AG administration group (6, AG 40 mg/kg, NAG), model group (6, LPS 100 μg/kg, M) and high dose (6, LPS 100 μg/kg + AG 40 mg/kg, AG) groups. *p < 0.05 or **p < 0.01 versus Normal group and #*p* < 0.05 or ##*p* < 0.01 versus Model group by ANOVA.

As shown in Figures 8A–D, after intravenous injection of LPS, serum progesterone of female rats increased significantly, which has potent anti-inflammatory effects. After prophylactic intragastric administration of AG, LPS injection did not increase progesterone. Additionally, there was no discernible change in blood progesterone levels when rats received AG alone without receiving an injection of LPS. Similar to testosterone, serum estradiol increased after LPS injection, and with prophylactic AG treatment, it tended to be the same as the normal group. Administration of AG alone without LPS injection did not decrease or increase serum levels of these hormones in female rats. Serum total testosterone levels did not differ significantly within each group due to large intra-group variability in the data, but there was a similar trend to the other hormones described above.

### 3.5 Effect of AG on TNF-α, IL-6, and IL-1β concentrations in serum

Figures 8E–G demonstrates that after receiving LPS, female rats’ blood concentrations of TNF-α, IL-6, and IL-1β were considerably higher than those in the normal group. By administering AG as a preventative measure, the amounts of these inflammatory cytokines were brought down to normal levels. However, serum TNF-α, IL-6, and IL-1β concentrations in non-LPS-stimulated rats, as well as the impact of AG on sex hormone levels, did not change substantially from the normal group in comparison.

### 3.6 Quantitative Real time polymerase chain reaction analysis for key enzymes involved in steroid hormone synthesis pathway

As shown in Figures 8H,I, StAR and CYP11A1, which are involved in steroid hormone synthesis, showed significant upregulation in the ovaries after LPS injection. The gene expression of both enzymes was downregulated in the AG-treated group compared to the model group. The data revealed a small elevation of StAR and CYP11A1 expression in the non-LPS-stimulated rats treated with AG, but no appreciable difference from the control group.

## 4 Discussion

In the present study, we found that the levels of several lipids in the serum of female rats were significantly increased following LPS-induced inflammation, which included 12 PC (phosphatidylcholine), PE (phosphatidylethanolamine), PS (phosphatidylserine), PG (phosphatidylglycerol), PI (phosphatidylinositol), PA (phosphatidic acid) or their hydrolysis products. However, such changes are not sex-specific. In previous work by our team, numerous markers of phospholipid metabolism were also identified in the serum of male rats (Zou, et al., 2017). Recent lipidomic research has further supported the idea that inflammation brought on by LPS stimulation can result in visibly altered lipid metabolism in the plasma or in tissues. (Shan, et al., 2019; Hahnefeld, et al., 2021).

Enrichment analysis showed that these metabolic markers were enriched in glycerophosphate, glycerophosphate metabolism and phosphatidylinositol metabolism pathway. Wang et al. (2020) applied a HPLC-QTOF/MS method to screen metabonomic changes in the serum, lung, bronchoalveolar lavage fluid, spleen and feces of male rats with intratracheal instillation of LPS to make acute lung injury (ALI). The result showed that ALI mainly also alters the metabolic pathways of glycerophospholipids, sphingolipids, linoleic acid (Wang, et al., 2020). Similarly, He L et al. reported that changes in plasma metabolites is mainly focused on sphingolipid**,** retinol and tryptophan metabolism pathways in ALI male rats (Hu et al., 2020).

Two factors may contribute to the abnormal phospholipid metabolism caused by LPS. One is that LPS alters the levels and functions of a number of phospholipid-related enzymes. The other is excessive oxidative stress caused by LPS. Serine palmitoyltransferase (SPT) is the first rate-limiting enzyme in sphingolipid synthesis, catalyzing the condensation of serine with palmitoyl-CoA. LPS increases the mRNA expression and activity of SPT, thereby stimulating two kinds of sphingolipids synthesis, ceramide, and sphingomyelin (Memon, et al., 1998). LPS also greatly increases the concentration of secretory phospholipase A2 in plasma (Dinkla, et al., 2016). Secretory phospholipase A2 catalyzes the hydrolysis of glycerophospholipids, which form the outer membrane of cell membranes, into lysophospholipids and free fatty acids. In addition, LPS attacks on cells produce an excess of reactive oxygen species (ROS), which oxidize the cell membrane to produce more free phospholipids (Raetz, et al., 2006). However, as described above, abnormalities in lipid metabolism and oxidative stress-related signalling pathways were found in both male and female rats following LPS stimulation.

In our metabolomic studies, an increase in progesterone and a kind of bile acid derivative--taurallocholic acid, was found after LPS stimulation. In metabolomic studies on LPS-stimulated inflammation models, the steroidal substances, such as pregnenolone (Hu, et al., 2020 **)**, calcitriol (Li, et al., 2020), and cholesterol sulfate (Wang, et al., 2020), were also found to be significantly increased in serum. An important cause of sex hormone and bile acids levels during LPS stimulation may be the elevation of cholesterol synthesis. The source of biosynthesis of sex hormone and bile acids *in vivo* is derived from cholesterol. Bile acids are metabolized by cholesterol in the liver by metabolic enzymes such as CYP7A1 and CYP27A1 (Björkhem and Eggertsen., 2001; Russell., 2003).

The literature reports that not only LPS, but also various cytokines such as TNF-α, IL-6, and IL-1β, promote elevated serum levels of cholesterol (Feingold, et al., 1993; Feingold and Grunfeld., 1987; Feingold, et al., 1991; Nonogaki, et al., 1995). This may be due to the fact that LPS boosts the transcription rate, protein quality and activity of HMG-CoA reductase, which in turn stimulates hepatic cholesterol synthesis (Feingold, et al., 1993). Like LPS, both TNF and IL-1 can stimulate *ad libitum* hepatic cholesterol by increasing HMG-CoA reductase activity and mRNA expression (Feingold, et al., 1993).

**FIGURE 9:**
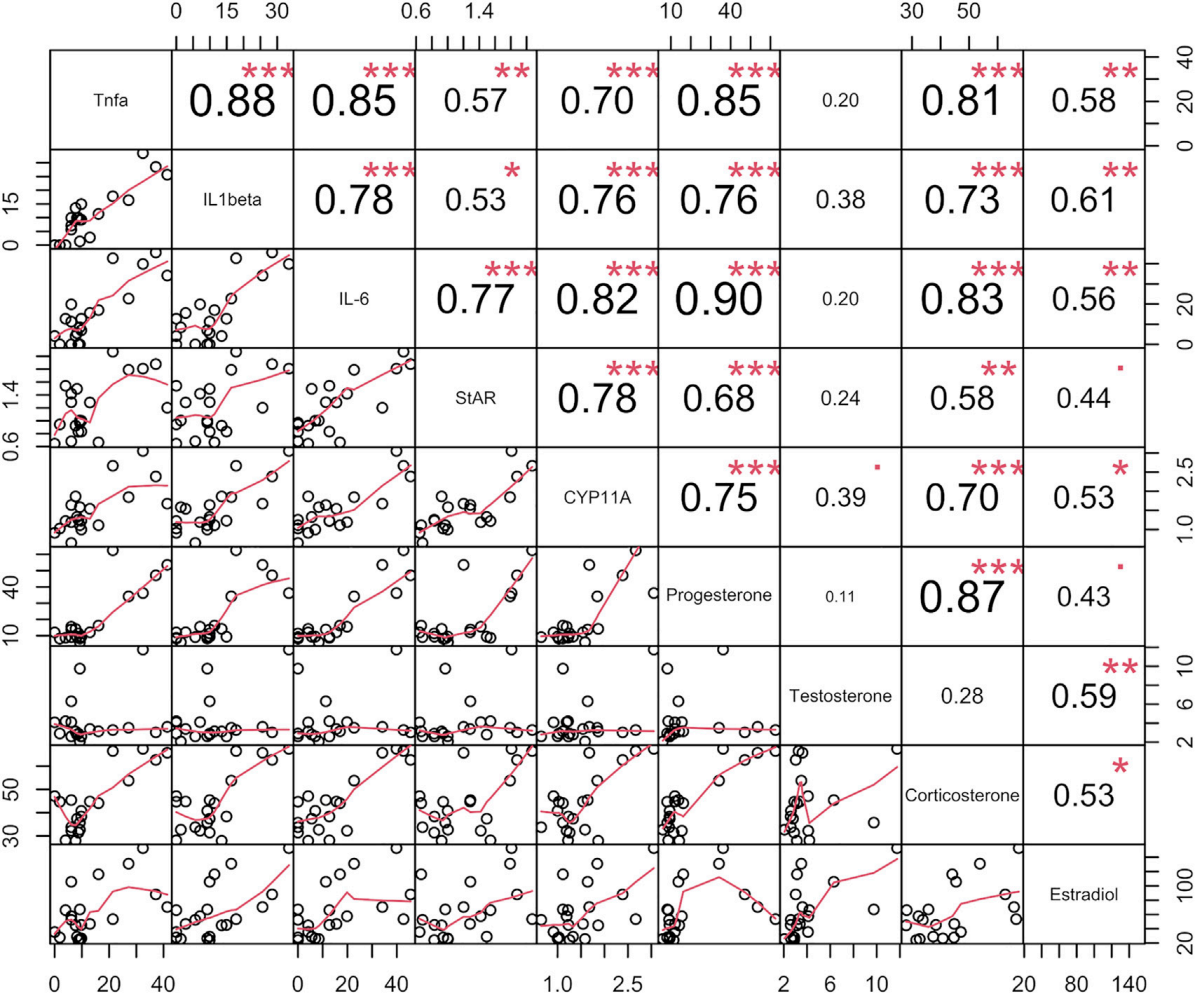
Correlation analysis of four sex steroids (progesterone, testosterone, estradiol and corticosterone), three inflmmatory cytokins (TNF-α, IL-6, and IL-1β) concentrations in serums, and the two sex hormone synthesis gene expression in ovories in verification experiment. The data name appears on the primary diagonal line. Data for each type is an aggregate of data of rats in all groups, including normal group (6, N), normal with AG administration group (6, AG 40 mg/kg, NAG), model group (6, LPS 100 μg/kg, M) and high dose (6, LPS 100 μg/kg + AG 40 mg/kg, AG) groups. The circles in the sub-boxes below the main diagonal indicate pairs of two kinds data of the same rat, and the red line depicts the correlation trend. The correlation coefficient is indicated in each box above the main diagonal.

Along with promoting the synthesis of cholesterol--the raw material for sex hormones, LPS also activates the hypothalamic-pituitary-adrenal axis (Beishuizen and Thijs., 2003). LPS contributes to hypothalamic activation through cytokines such as IL-1β, which upregulates ACTH levels, thereby contributing to the production of hormones such as cortisol by the adrenal glands. Correlation analysis of cytokine and sex-related hormone levels (Figure 9) showed a significant positive correlation following LPS-inflammation.

Although endotoxin exerts an inhibitory effect on the hypothalamic-pituitary-gonadal axis, downregulating GnRH levels (Barabás, et al., 2020), LPS contribute to the expression and activity of a variety of enzymes that convert cholesterol to sex hormones in the ovary (Yoo and Lee., 2016). These hormones are all powerfully anti-inflammatory. This is a classic negative feedback to maintain homeostasis.

Steroid hormone synthesis begins with the conversion of cholesterol to pregnenolone, with the first rate-limiting enzymes being steroidogenic acute regulatory protein (StAR) and cytochrome P450scc enzyme (CYP11A1) (Miller and Auchus., 2011). Pregnenolone is then converted to sex steroid hormones including progesterone, androstenedione, testosterone and estradiol in turn (Miller and Auchus., 2011). After endotoxin challenge, it was found that females showed higher levels of sex hormone synthesis. Intraperitoneal injection of LPS at 1.5 mg/kg resulted in increased serum corticosterone, progesterone and total testosterone levels in females, whereas males were only found to have decreased total testosterone concentrations, with no change in other steroid hormone levels (Kosyreva,et al., 2018). Our results also showed that LPS markedly increased the serum levels of corticosterone, estradiol, and progesterone in inflammatory female rats. The results of qRT-PCR showed that LPS increased the expression of StAR and CYP11A1 in a stressed manner.

**FIGURE 10:**
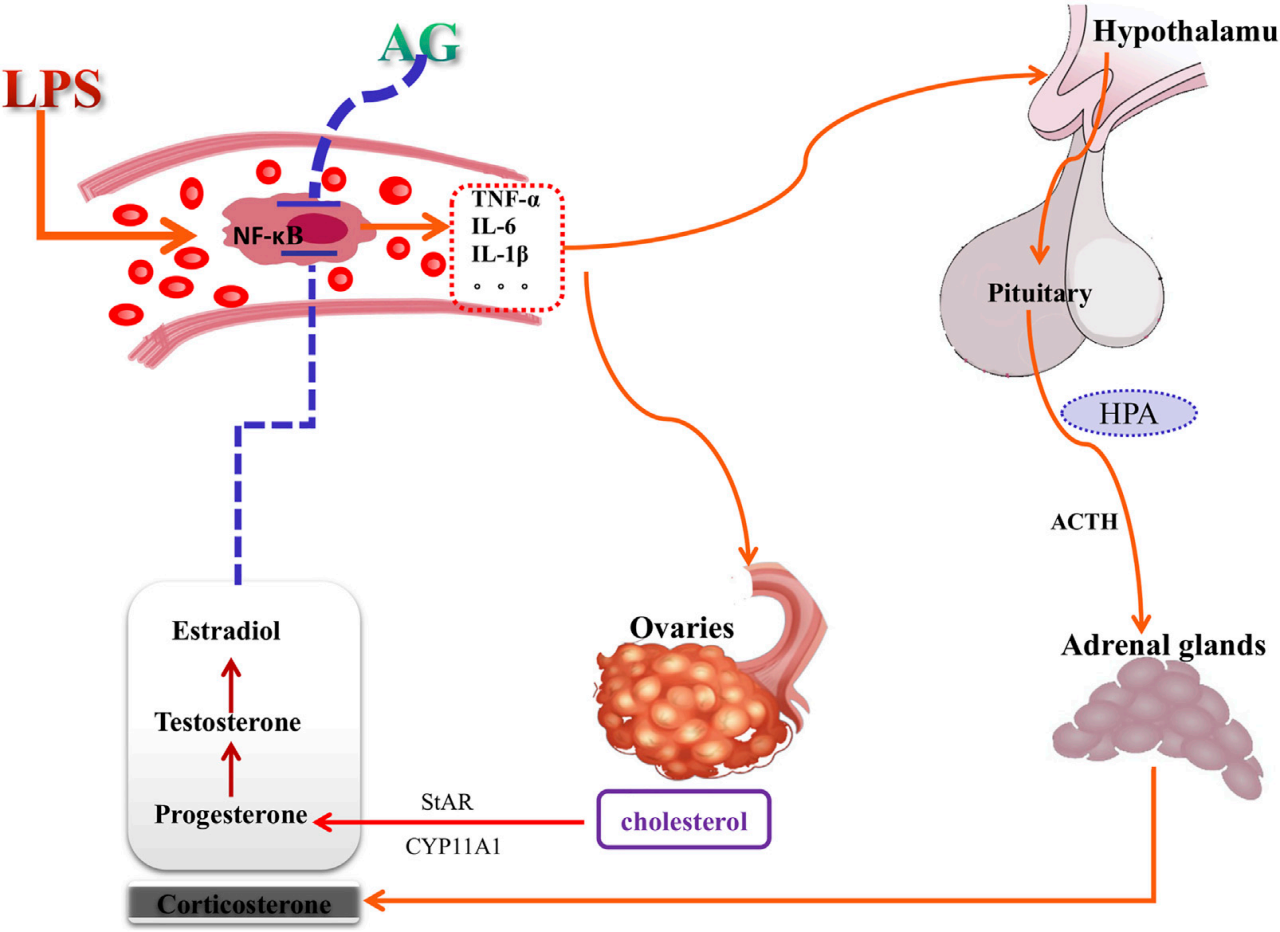
The mechanism of andrographolide (AG) acting on sex hormone production pathways. Red arrows indicate stimulation and blue vertical bars indicate inhibition. AG acting on sex hormone production may be mediated by anti-inflammatory action, and is an indirect attributes.

In post-inflammatory female rats, the results showed that AG decreased sex hormone levels and prevented the upregulation of StAR and CYP11A1. However, AG had no appreciable impact on the hormone concentrations and gene expression in non-inflamed female rats. Similarly, AG reduced TNF-α, IL-6 and IL-1β production in LPS-induced rats but did not change these inflammatory factors’ concentrations in non-inflammatory rats. AG inhibits a variety of LPS-induced inflammatory cascade signaling pathways, including the phosphorylation of IKKβ, IκBα, and NF-κB and JNK (Zhu et al., 2013; Wong et al., 2016). Therefore, administration of AG resulted in a significant reduction in cytokine production during inflammation. As mentioned above, LPS and many inflammatory cytokins stimulate the synthesis of hormones, and these released hormones can inhibit the aggravation and deterioration of inflammation, which is a typical negative feedback mechanism of anti-inflammation. AG significantly inhibits the production of cytokines, thereby removing inducers of sex hormone production. In conclusion, considering the knowledge of the biology of the interaction between LPS and sex hormones and the observation that AG does not inhibit or promote sex hormones in non-inflamed female rats, it can be assumed that the inhibitory effect of AG on sex hormones during inflammation is mediated through its inhibition of the synthesis of inflammatory cytokines. Through the correlation analysis of cytokines, sex hormone and invertase levels (Figure 9), and combined with the analysis of the literature of AG, Figure 10 summarizes the possible mechanism of AG’s action on the production of sex hormone. It shows that the inhibitory effect of AG on the production of sex hormone in the inflammatory process is indirectly exerted by reducing inflammatory factors first, and then blocking the stimulation of these factors on hormone synthesis.

Although metabolomics can identify potential targets of drug action through metabolites (Johnson et al., 2016), the targets it suggests are the results of both drug activity and illness changes. A drug can either directly interfere with metabolite production or indirectly alter the commencement or termination of metabolite synthesis pathways via other mechanisms. In other words, a drug’s impact on metabolites in a disease state can be either direct or indirect. Consequently, to determine the mode of effect of drugs on metabolites, comprehensive analysis needs to be conducted in combination with biological experiments or prior biological knowledge.

## 5 Conclusion

After prophylactic treatment of andrographolide, the metabolic pathways in LPS-induced female rats, including steroid hormone, glycerophosphate, glycerolipids, and inositol phosphate synthesis pathways, were markedly reversed. Andrographolide suppressed upregulation of StAR and CYP11A1 and then decreased sex hormone in post-inflammatory females rats, whereas andrographolide had no effect on these in non-inflamed female rats.

## Data Availability

The original contributions presented in the study are included in the article/supplementary material, further inquiries can be directed to the corresponding authors.
